# Correction to: *PyPlr*: A versatile, integrated system of hardware and software for researching the human pupillary light reflex

**DOI:** 10.3758/s13428-022-01937-x

**Published:** 2022-08-24

**Authors:** Joel T. Martin, Joana Pinto, Daniel Bulte, Manuel Spitschan

**Affiliations:** 1 Department of Engineering Science, Institute of Biomedical Engineering, University of Oxford, Oxford, OX3 7DQ UK; 2 Department of Experimental Psychology, University of Oxford, Oxford, OX2 6GG UK; 3 Department of Psychology, University of York, York, YO10 5DD UK; 4 TUM Department of Sport and Health Sciences (TUM SG), Technical University of Munich, Munich, Germany; 5 Max Planck Institute for Biological Cybernetics, Tübingen, Germany

**Correction to: Behav Res**

10.3758/s13428-021-01759-3

The authors report changes to Fig, 1 of this paper (below). ORCIDs and affiliations have also been updated.





